# RCN1 suppresses ER stress-induced apoptosis via calcium homeostasis and PERK–CHOP signaling

**DOI:** 10.1038/oncsis.2017.6

**Published:** 2017-03-20

**Authors:** S Xu, Y Xu, L Chen, Q Fang, S Song, J Chen, J Teng

**Affiliations:** 1 Key Laboratory of Cell Proliferation and Differentiation of the Ministry of Education, State Key Laboratory of Bio-membrane and Membrane Bio-engineering, College of Life Sciences, Peking University, Beijing, China; 2 Center for Quantitative Biology, Peking University, Beijing, China

## Abstract

Endoplasmic reticulum (ER) stress is caused by the disturbance of ER homeostasis and leads to the activation of the unfolded protein response (UPR), which alleviates stress at an early stage and triggers apoptosis if homeostasis fails over a prolonged timeframe. Here, we report that reticulocalbin 1 (RCN1), a member of the CREC family, is transactivated by nuclear factor kappa B (NF-κB) during ER stress and inhibits ER stress-induced apoptosis. The depletion of RCN1 increases the UPR during drug-induced ER stress by activating PRKR-like ER kinase–CCAAT/enhancer-binding protein-homologous protein (PERK–CHOP) signaling, thus inducing apoptosis. Furthermore, we found that the first two EF-hand calcium-binding motifs of RCN1 specifically interact with inositol 1,4,5-trisphosphate (IP_3_) receptor type 1 (IP_3_R1) on loop 3 of its ER luminal domain and inhibit ER calcium release and apoptosis. Together, these data indicate that RCN1, a target of NF-κB, suppresses ER calcium release by binding to IP_3_R1 and decreases the UPR, thereby inhibiting ER stress-induced apoptosis.

## Introduction

The endoplasmic reticulum (ER), a dynamic sheet and tubular organelle, has multiple functions including initial protein maturation, lipid synthesis and maintenance of intracellular calcium homeostasis.^1, 2^ Disturbance of the ER environment can cause ER stress and trigger the activation of a signaling network termed the unfolded protein response (UPR), which transduces information about protein folding status in the ER lumen to the cytosol and nucleus. There are three major ER membrane transducers: PRKR-like ER kinase (PERK),^3^ activating transcription factor 6 (ATF6)^4^ and inositol-requiring kinase-1 (IRE1).^5^ The activation of these proteins alleviates ER stress by inhibiting protein transcription, expanding the ER membrane and elevating chaperone levels.^6^ However, when cells experience irreversible ER stress, PERK and IRE1 signaling eliminate damaged cells by apoptosis.^7^ During apoptosis, IRE1 recruits tumor necrosis factor alpha (TNF-α) receptor associated factor 2 and apoptosis signal regulating kinase-1, thus culminating in activation of c-Jun N-terminal protein kinase.^8, 9^ PERK activation leads to phosphorylation of eukaryotic translation initiation factor 2α (eIF2α) and selectively induces activating transcription factor 4 (ATF4), a transcription factor that enhances the expression of pro-apoptotic CCAAT/enhancer-binding protein-homologous protein (CHOP).^10, 11^ CHOP subsequently downregulates anti-apoptotic BCL-2, induces pro-apoptotic Bim, upregulates DNA damage-inducible 34 (GADD34) and finally contributes to apoptosis.^12, 13^

Calcium, a highly dynamic and versatile intracellular signal, is stored primarily in the ER.^14^ The storage of calcium in the ER is regulated by three types of proteins: (i) Ca^2+^ pumps, which transport Ca^2+^ from the cytosol to the ER; (ii) Ca^2+^-binding proteins, such as calcium buffer proteins and calcium sensors; and (iii) Ca^2+^ channels, which release Ca^2+^ to the cytosol.^15^ Dysfunction of these proteins leads to alterations in ER calcium homeostasis, including ER calcium depletion, and ultimately results in ER stress.^16^ Inositol 1,4,5-trisphosphate (IP_3_) receptor (IP_3_R), one of the primary ubiquitous intracellular Ca^2+^ release channels, has three isoforms (IP_3_R1, IP_3_R2 and IP_3_R3). These isoforms are encoded by different genes but share 60–80% homology with respect to their amino acid sequences.^17^ These proteins form tetrameric Ca^2+^ channels that are regulated by multiple factors, including Ca^2+^ concentration in the cytosol and the ER, IP_3_, ATP, phosphorylation status and interacting proteins.^18^ During apoptosis, the activation of IP_3_R increases cytosolic Ca^2+^ concentrations and results in excessive Ca^2+^ accumulation in the mitochondria, thereby increasing mitochondrial membrane permeabilization and release of cytochrome *c*.^19, 20^

Reticulocalbin 1 (RCN1), a member of the CREC family, is encoded by the *RCN1* gene. To date, only one spliced isoform has been reported; this isoform contains six EF-hand motifs (calcium-binding motifs) and an HDEL sequence, an ER retention signal, on its C terminus.^21^ An increasing number of reports indicate upregulation of RCN1 in cancer patients and multiple tumor types, including breast cancer,^22^ colorectal cancer,^23^ kidney cancer^24^ and liver cancer,^25^ but the underlying mechanism remains unclear. Here, we report that RCN1, regulated by nuclear factor kappa B (NF-κB), suppresses ER stress-induced UPR signaling and apoptosis by inhibiting IP_3_R1-mediated ER calcium release.

## Results

### RCN1 inhibits ER stress-induced apoptosis

**Figure 1:**
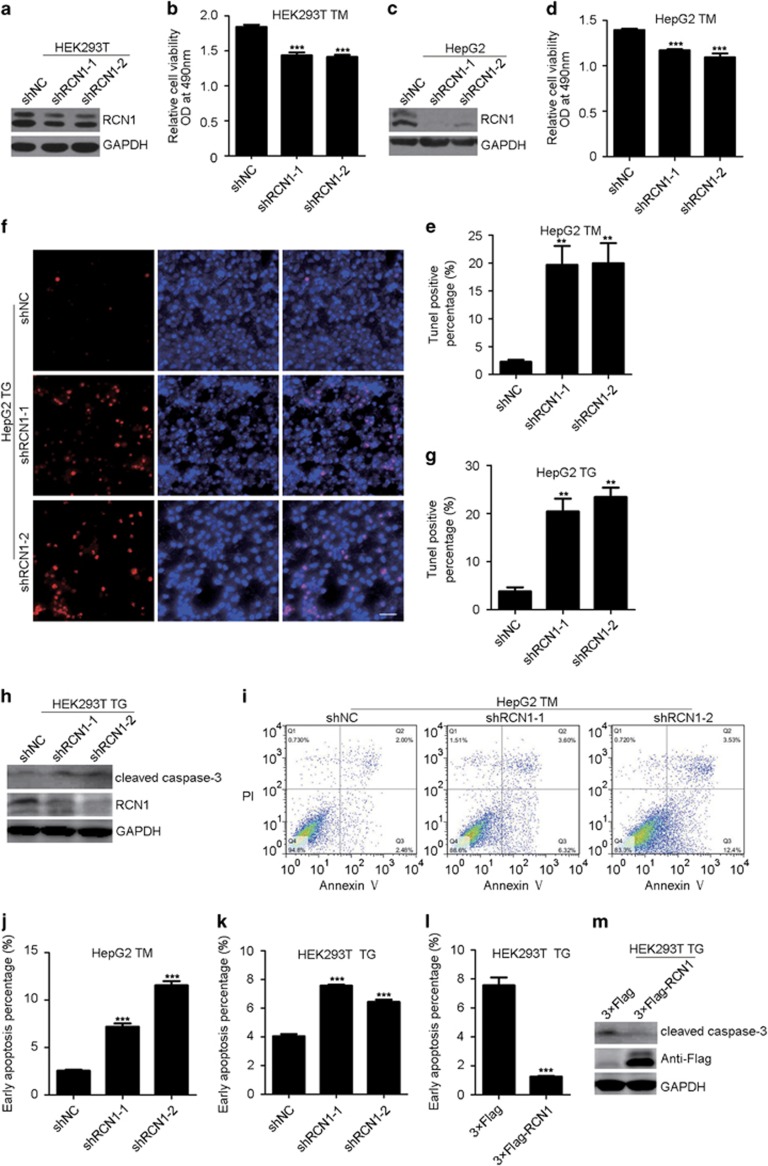
RCN1 inhibits ER stress-induced apoptosis. (**a**) Western blots of RCN1-knockdown efficiency in HEK293T cells. NC, negative control. (**b**) MTS assay of viable HEK293T cells expressing shNC, shRCN1-1 or shRCN1-2 vectors treated with TM (2 μm) for 48 h (*n*=3). (**c**) Western blots of control and RCN1-knockdown HepG2 cells. (**d**) MTS assay of viable HepG2 cells expressing shNC, shRCN1-1 or shRCN1-2 vectors treated with TM (8 μm) for 48 h (*n*=3). (**e**) Quantification of TUNEL-positive control or RCN1-knockdown HepG2 cells treated with TM (8 μm) for 36 h (*n*=3; >100 cells per experiment). (**f**) Representative images from TUNEL assays of apoptotic control or RCN1-knockdown HepG2 cells treated with TG (4 μm) for 36 h. Scale bar, 50 μm. (**g**) Quantification of TUNEL-positive cells in **f** (*n*=3; >100 cells per experiment). (**h** and **m**) Western blots of cleaved caspase-3 in RCN1-knockdown (**h**) and RCN1-overexpressing (**m**) HEK293T cells after treatment with TG (1 μm). (**i**) Flow cytometry analysis of apoptotic cells labeled by annexin V and PI in TM (8 μm, 36 h)-treated control or RCN1-knockdown HepG2 cells. (**j**) Quantification of apoptotic cells in **i** (*n*=3; 10 000 cells per experiment). (**k**) Quantification of early apoptotic cells labeled by annexin V and PI in TG (4 μm, 36 h)-treated control or RCN1-knockdown HEK293T cells (*n*=3; 10 000 cells per experiment). (**l**) Quantification of early apoptotic cells labeled by annexin V and PI in TM (2 μm, 36 h)-treated control or RCN1-overexpressing HEK293T cells (*n*=3; 10 000 cells per experiment). For **b**, **d**, **e**, **g** and **j**–**l,** data are presented as the mean±s.e.m. **P<0.01, ***P<0.001, as determined by unpaired two-tailed Student’s *t*-test.

To investigate the function of RCN1 in ER stress, we first determined the effect of RCN1 on cell survival after treatment with the ER stress-inducing drug tunicamycin (TM). The viability of cells was assessed by microscopy and cell proliferation assays (MTS assay), and the results indicated that depletion of RCN1 resulted in a lower number of surviving HEK293T and HepG2 cells after TM treatment (Figures 1a and d, Supplementary Figure S1a). To further investigate whether RCN1 promotes cell survival by inhibiting apoptosis, we performed terminal deoxynucleotidyl transferase dUTP nick end-labeling (TUNEL) assays in TM-treated HepG2 cells. Depletion of RCN1 dramatically increased the number of TUNEL-labeled cells (Figure 1e, Supplementary Figure S1b); similar results were also observed after treatment with TG (thapsigargin, another ER stress-inducing drug)-treated HepG2 cells (Figures 1f and g). Furthermore, a distinct cleaved caspase-3 band appeared in TG-treated RCN1-knockdown HEK293T cells (Figure 1h) but not in untreated RCN1-knockdown HepG2 and HEK293T cells (Supplementary Figure S1c and d), thus indicating that RCN1 depletion promotes ER stress-induced apoptosis. Annexin V/PI (propidium iodide) labeling assays revealed that depletion of RCN1 during ER stress, but not under normal conditions, significantly elevated the number of cells in early apoptosis, as detected by flow cytometry analysis (Figures 1i–k, Supplementary Figure S1e–g), whereas overexpression of 3 × Flag-RCN1 decreased the number of cells in early apoptosis and the cleaved caspase-3 levels during ER stress (Figures 1l and m, Supplementary Figure S1h) but not under normal conditions (Supplementary Figure S1i–k). Together, these results demonstrate that RCN1 inhibits ER stress-induced apoptosis.

### RCN1 inhibits PERK- and IRE1-mediated UPR signaling during ER stress

**Figure 2:**
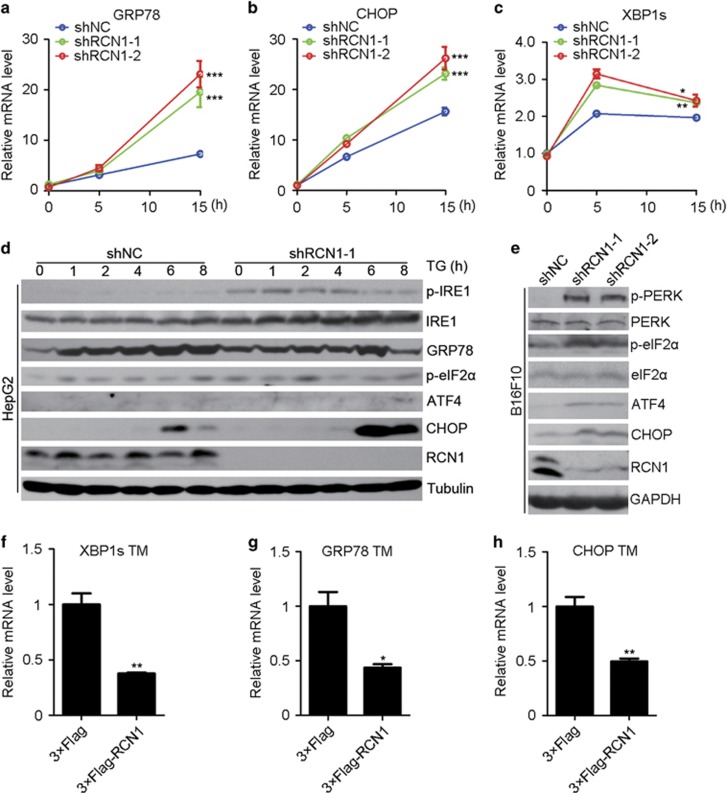
RCN1 inhibits UPR signaling. (**a**–**c**) Quantitative real-time PCR of the relative mRNA expression levels of GRP78 (**a**), CHOP (**b**) and XBP1s (**c**) in the negative control (shNC) and RCN1-knockdown (shRCN1) HepG2 cells after treatment with TM (8 μm) at the indicated time points. (**d**) Western blots of p-IRE1, IRE1, GRP78, p-eIF2α, ATF4 and CHOP in control and RCN1-knockdown HepG2 cells after treatment with TG (4 μm) at the indicated time points. (**e**) Western blots of p-PERK, PERK, p-eIF2α, eIF2α, ATF4 and CHOP in control and RCN1-knockdown B16F10 cells after treatment with TG (4 μm). (**f**–**h**) Quantitative real-time PCR of relative mRNA expression levels of XBP1s (**f**), GRP78 (**g**) and CHOP (**h**) in control (3 × Flag) and 3 × Flag-RCN1-overexpressing HEK293T cells after TM (2 μm, 8 h) treatment. For **a**–**c** and **f**–**h**, data are presented as the mean±s.e.m. *P<0.05, **P<0.01, ***P<0.001, as determined by unpaired two-tailed Student’s *t*-test.

To determine whether RCN1 inhibits UPR signaling, we performed quantitative real-time PCR (RT–PCR) and measured the transcription levels of UPR-related genes. The messenger RNA (mRNA) levels of GRP78 and CHOP, two major markers of UPR signaling, were significantly increased in RCN1-knockdown HepG2 cells after TM treatment (Figures 2a and b), whereas XBP1s, a spliced form of XBP1 that is cleaved by IRE1,^26^ exhibited a relatively smaller increase (Figure 2c). Furthermore, in these cells, we measured the expression levels of UPR target genes, such as p58^ipk^, Herp, ERdj4, ATF4, EDEM1, and WARS,^7^ ATF4 and p58^ipk^, which function downstream of PERK signaling, exhibited expression patterns similar to those of CHOP (Figure 2b, Supplementary Figure S2a). These data suggest that RCN1 regulates the PERK- and IRE1-mediated pathways after TM treatment.

To verify that RCN1 participates in the IRE1- and PERK-mediated pathways, we measured phosphorylated IRE1 (p-IRE1) and PERK (p-PERK), the active forms of IRE1 and PERK, respectively. The levels of p-IRE1 increased significantly 2 h after TG treatment in RCN1-knockdown HepG2 cells but decreased in control cells (Figure 2d). We also observed an increase in the protein level of p-PERK in RCN1-knockdown B16 cells, as well as an increase in the levels of p-eIF2a, ATF4 and CHOP in RCN1-knockdown HepG2 and B16F10 cells (Figures 2d and e) during ER stress, whereas cleaved ATF6 exhibited no changes (Supplementary Figure S2b). Furthermore, in the RT–PCR analysis, overexpression of 3 × Flag-RCN1 decreased the mRNA levels of XBP1s, GRP78 and CHOP in HEK293 cells after TM and TG treatment (Figures 2f and h, Supplementary Figures S2c-e). Collectively, these data indicate that RCN1 inhibits PERK- and IRE1-mediated UPR signaling during ER stress.

### RCN1 inhibits ER stress-induced apoptosis via PERK–CHOP signaling

**Figure 3:**
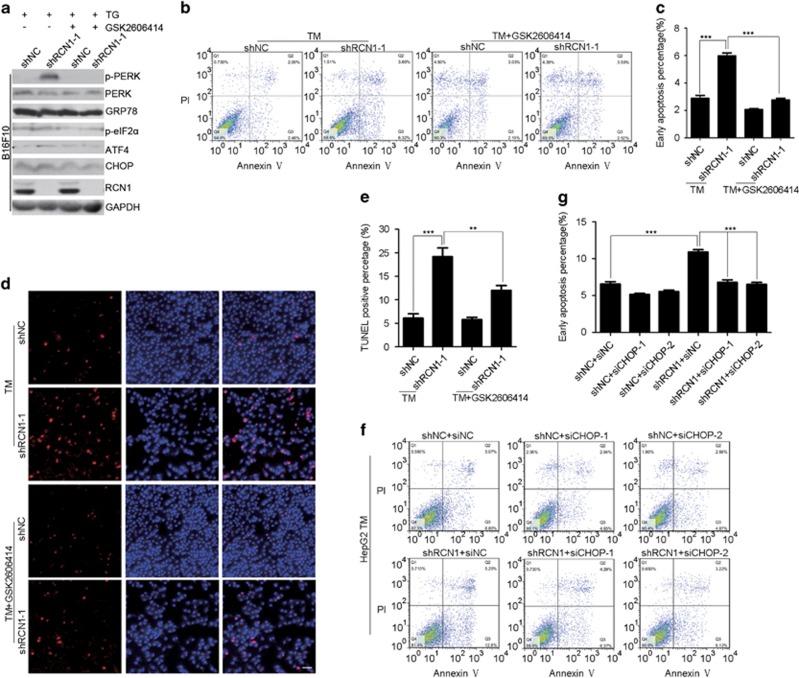
RCN1 depletion enhances ER stress-induced apoptosis via activation of PERK–CHOP signaling. (**a**) Western blots of p-PERK, PERK, GRP78, p-eIF2α, ATF4, CHOP and RCN1 in the negative control (shNC) and RCN1-knockdown (shRCN1) B16F10 cells treated with TG (5 μm, 16 h) in the presence or absence of GSK2606414 (2 μm, 1 h) treatment. (**b**) Flow cytometry analysis of apoptotic cells labeled by annexin V and PI in TM-treated (8 μm, 36 h) control or RCN1-knockdown HepG2 cells in the presence or absence of GSK2606414 (2 μm, 1 h) treatment. (**c**) Quantification of early apoptotic cells in **b** (*n*=3; 10 000 cells per experiment). (**d**) Representative images from a TUNEL assay of labeled apoptotic cells in control and RCN1-knockdown HepG2 cells treated with TM (8 μm, 48 h) in the presence or absence of GSK2606414 (2 μm, 1 h) treatment. Scale bar, 50 μm. (**e**) Quantification of TUNEL-positive cells in **d** (*n*=3; >100 cells per experiment). (**f**) Flow cytometry analysis of apoptotic cells labeled by annexin V and PI in siNC, siCHOP-1 or siCHOP-2 transfected control and RCN1-knockdown HepG2 cells after TM (8 μm, 48 h) treatment. (**g**) Quantification of early apoptotic cells in (**f**) (*n*=3; 10 000 cells per experiment). For **c**, **e** and **g**, the data are presented as the mean±s.e.m. **P<0.01, ***P<0.001, as determined by unpaired two-tailed Student’s *t*-test.

To investigate whether the PERK- or IRE1-mediated pathways contribute to increased apoptosis caused by RCN1 depletion during ER stress, we used inhibitors of these transducers. Pretreatment with GSK2606414, a PERK inhibitor,^27^ efficiently blocked the CHOP induction caused by depletion of RCN1 after TG treatment (Figure 3a). Moreover, flow cytometry analysis showed that inhibition of PERK activation significantly alleviated the increased ratio of early apoptotic cells in RCN1-knockdown cells after TM treatment (Figures 3b and c), whereas inhibition of IRE1 activation by STF883010^28^ resulted in no change (Supplementary Figures S3a and b), thus indicating that PERK, but not IRE1, promotes apoptosis in response to RCN1 depletion during ER stress. Furthermore, a TUNEL assay confirmed that inhibition of PERK activation resulted in a significant decrease in the number of apoptotic cells after depletion of RCN1 during ER stress (Figures 3d and e). To further confirm that the PERK-mediated signaling pathway is involved in increased apoptosis caused by RCN1 depletion during ER stress, we selected efficient small interfering RNAs (siRNAs) targeting CHOP (Supplementary Figure S3c) and determined that depletion of CHOP in RCN1-knockdown HepG2 cells also mitigated the increased early apoptosis after treatment with TM (Figures 3f and g). Together, these data demonstrate that RCN1 inhibits ER stress-induced apoptosis through the PERK–CHOP signaling pathway.

### RCN1 interacts with IP_3_R1 and inhibits ER calcium release

**Figure 4:**
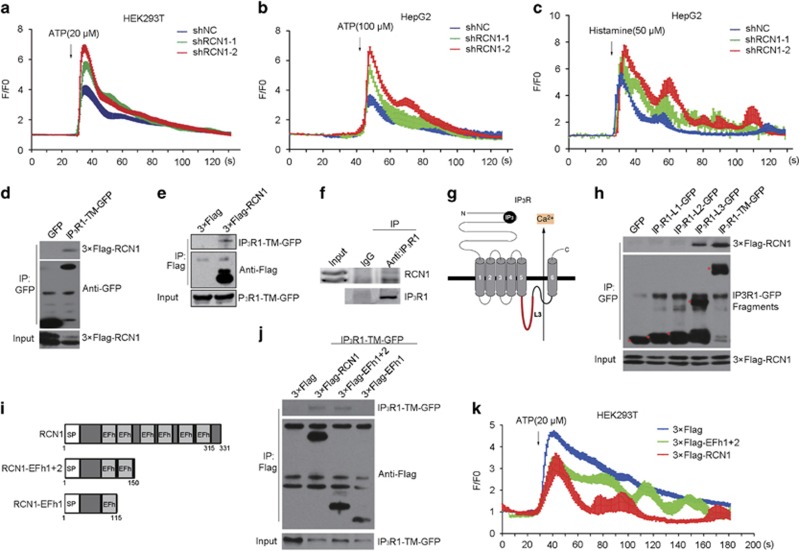
RCN1 interacts with IP_3_R1 and inhibits ER calcium release. (**a**) Representative Ca^2+^ traces of cytosolic Ca^2+^ after treatment with ATP (20 μm) in negative control (shNC) or RCN1 shRNA-transfected (shRCN1) HEK293T cells. (**b** and **c**) Representative Ca^2+^ traces of cytosolic Ca^2+^ after treatment with ATP (100 μm) (**b**) or histamine (50 μm) (**c**) in negative control or RCN1-knockdown HepG2 cells. (**d**) HEK293T cells co-transfected with 3 × Flag-RCN1 and GFP or IP_3_R1-TM (transmembrane domain)-GFP were subjected to immunoprecipitation (IP) using anti-GFP antibody. The immunoprecipitates were immunoblotted with anti-Flag or anti-GFP antibody. (**e**) HEK293T cells co-transfected with IP_3_R1-TM-GFP and 3 × Flag or 3 × Flag-RCN1 were subjected to immunoprecipitation using anti-Flag antibody. The immunoprecipitates were immunoblotted with anti-Flag or anti-GFP antibody. (**f**) HepG2 cells were subjected to immunoprecipitation using anti-IP_3_R1 antibody. The immunoprecipitates were immunoblotted with anti-RCN1 or anti-IP_3_R1 antibody. (**g**) Schematic of IP_3_R1 structure. (**h**) Mapping of the domains of IP_3_R1 required for interaction with RCN1. HEK293T cells co-overexpressing GFP-tagged IP_3_R1 truncations (IP_3_R1-L1-GFP, IP_3_R1-L2-GFP, IP_3_R1-L3-GFP) and 3 × Flag-RCN1 were subjected to immunoprecipitation with anti-GFP antibody. The immunoprecipitates were immunoblotted with anti-Flag or anti-GFP antibody. (**i**) Schematic of RCN1 truncation constructs. SP, signal peptide; EFh, EF-hand. (**j**) HEK293T cells co-transfected with IP_3_R1-TM-GFP and 3 × Flag, 3 × Flag-RCN1, 3 × Flag-RCN1-EFh1+2, or 3 × Flag-RCN1-EFh1 were subjected to immunoprecipitation using anti-Flag antibody. The immunoprecipitates were immunoblotted with anti-Flag or anti-GFP antibody. (**k**) Basal Ca^2+^ level tracked by fluo-4 in ATP-treated HEK293T cells transfected with control, 3 × Flag-RCN1 or 3 × Flag-EFh1+2. Representative Ca^2+^ traces of cytosolic Ca^2+^ after treatment with ATP (100 μm) in control (3 × Flag)-, 3 × Flag-EFh1+2- or 3 × Flag-RCN1-transfected HEK293T cells.

Because ER calcium depletion is a major cause of ER stress,^13^ we sought to investigate whether RCN1 affects ER calcium release. A fluo-4 tracking assay demonstrated that the F/F0 ratio, which indicates the change in the free cytosolic calcium concentration, displayed no significant changes during general ER homeostasis in RCN1-knockdown cells; however, after ATP or histamine treatment, the F/F0 ratio was elevated in RCN1-knockdown HepG2 and HEK293T cells (Figures 4a–c), thus suggesting that RCN1 prevents ER calcium release after stimulation.

Because RCN1 resides in the ER lumen^21^ and IP_3_Rs are the major calcium release channel in the ER membrane,^18^ we investigated whether these proteins associate with each other. Immunoprecipitation revealed that exogenously expressed IP_3_R1-TM-GFP (GFP-tagged IP_3_R1 truncation mutants containing the transmembrane domain) specifically interacted with 3 × Flag-RCN1 (Figures 4d and e), whereas IP_3_R3-TM-GFP exhibited much less interaction (Supplementary Figure S4a). In addition, an endogenous immunoprecipitation assay further verified the interaction of RCN1 with IP_3_R1 (Figure 4f). We then generated a series of truncated or depletion mutants of IP_3_R1 and RCN1 to dissect the functional domains of the RCN1–IP_3_R1 interaction. The truncations, consisting of intra-luminal loop domains of IP_3_R1, exhibited the possibility of interacting with RCN1. An immunoprecipitation assay further revealed that only intra-luminal loop 3 of IP_3_R1 (IP_3_R1-L3-GFP) interacted with exogenous RCN1 (Figures 4g and h). We also generated a series of EF-hand depletion mutants of RCN1 to dissect the functional domains necessary for RCN1 interaction with IP_3_R1. The first two EF-hand depletion mutants of RCN1 were not able to interact with IP_3_R1 (Supplementary Figures S4b and c), whereas RCN1 truncation mutants containing the first two EF-hands (3 × Flag-RCN1-EFh1+2) interacted with IP_3_R1-TM-GFP (Figures 4i and j), thus confirming that the first two EF-hands of RCN1 are critical to the interaction with IP_3_R1.

Next, we determined whether the first two EF-hands of RCN1 are required for RCN1 function. We first assayed calcium release and found that 3 × Flag-RCN1-EFh1+2 partially inhibited calcium release relative to 3 × Flag-RCN1 in HEK293T cells after ATP treatment (Figure 4k). Therefore, the first two EF-hands of RCN1 can partially compensate for full-length RCN1, thus indicating that the interaction of RCN1 and IP_3_R1 is critical for the inhibition of IP_3_R1 activity.

RCN1 is a calcium-binding protein.^29^ Therefore, to investigate whether the interaction of RCN1 with IP_3_R1 is regulated by calcium concentration, we performed *in vitro* immunoprecipitation assays. Because free calcium concentrations decreased from 400 μM (free ER calcium concentration in physiological conditions) to 0 μM, the interaction of 3 × Flag-RCN1 and IP_3_R1-TM-GFP increased, thus suggesting that the RCN1–IP_3_R1 interaction is regulated by ER calcium concentration (Supplementary Figure S4d).

### RCN1 inhibits CHOP levels and apoptosis via IP_3_R1

**Figure 5:**
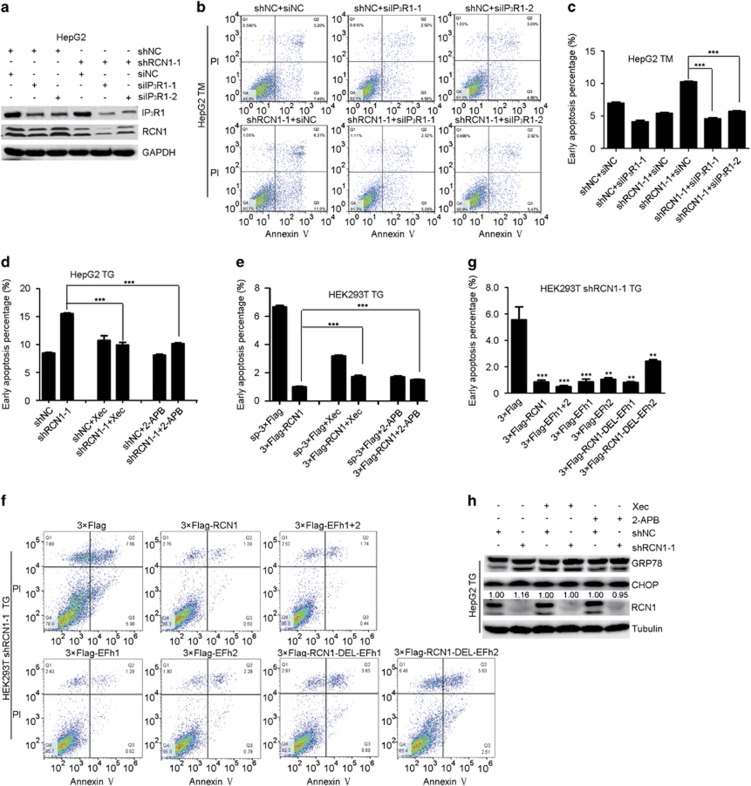
RCN1 inhibits CHOP-mediated apoptosis via IP_3_R1. (**a**) Western blots of IP_3_R1 knockdown efficiency in the negative control (shNC) and RCN1-knockdown (shRCN1) HepG2 cells. (**b**) Flow cytometry analysis of apoptotic cells labeled by annexin V and PI in control or RCN1-knockdown HepG2 cells in the presence or absence of IP_3_R1 knockdown and treatment with TM (8 μm). (**c**) Quantification of early apoptotic cells in **b** (*n*=3; 10 000 cells per experiment). (**d**) Quantification of early apoptotic cells labeled by annexin V and PI after TG treatment (4 μm) in the presence or absence of pretreatment with Xec (0.1 μm) or 2-APB (2 μm) in control or RCN1-knockdown HepG2 cells in a flow cytometry analysis. (**e**) Quantification of early apoptotic cells labeled by annexin V and PI after TG (4 μm) treatment in the presence or absence of pretreatment with Xec (0.1 μm) or 2-APB (2 μm) in control or RCN1-overexpressing HepG2 cells in a flow cytometry analysis. (**f**) Flow cytometry analysis of apoptotic cells labeled by annexin V and PI in RCN1-knockdown HEK293T cells transfected with control (3 × Flag), 3 × Flag-RCN1, 3 × Flag-EFh1+2, 3 × Flag-EFh1, 3 × Flag-EFh2, 3 × Flag-RCN1-DEL-EFh1 or 3 × Flag-RCN1-DEL-EFh2 after TG (5 μm, 24 h) treatment. (**g**) Quantification of early apoptotic cells in (**f**) (*n*=3; 10 000 cells per experiment). (**h**) Western blots of GRP78 and CHOP after TG (4 μm) treatment in the presence or absence of pretreatment with Xec (0.1 μm) or 2-APB (2 μm) in control or RCN1-knockdown HepG2 cells. The numbers indicate the relative expression levels of CHOP. For **c**–**e** and **g**, the data are presented as the mean±s.e.m. **P<0.01, ***P<0.001, as determined by unpaired two-tailed Student’s *t*-test.

To determine whether RCN1 inhibits apoptosis via IP_3_R1, we selected efficient siRNAs targeting IP_3_R1 (Figure 5a). Flow cytometry analysis demonstrated that depletion of IP_3_R1 significantly alleviated the increased number of early apoptotic cells caused by depletion of RCN1 after TM treatment (Figures 5b and c). In addition, flow cytometry analysis revealed that after TG treatment, inhibition of IP_3_R1 by 2-APB (2-aminoethoxydiphenyl borate)^30^ and Xec (Xestospongin C)^31^ significantly alleviated the increase in early apoptosis cells in RCN1-knockdown cells (Figure 5d, Supplementary Figure S5a), but not in RCN1-overexpressing cells (Figure 5e, Supplementary Figure S5b). Moreover, flow cytometry analysis indicated that overexpression of either of the first two EF-hands of RCN1 containing the IP_3_R1 binding domain significantly alleviated the number of cells in early apoptosis induced by RCN1 depletion during ER stress (Figures 5f and g), thereby indicating that RCN1 reduces apoptosis in an IP_3_R1-dependent manner.

Considering the connection between the UPR and apoptosis,^13^ we investigated whether IP_3_R1 participates in the augmented UPR signaling caused by RCN1 depletion. Western blots revealed that inhibition of IP_3_R1 alleviated the increased CHOP level induced by depletion of RCN1 after TG treatment (Figure 5h), thus suggesting that ER calcium disturbance caused by RCN1 depletion participates in PERK–CHOP signaling during ER stress.

### Expression of RCN1 is enhanced by NF-κB activation

**Figure 6:**
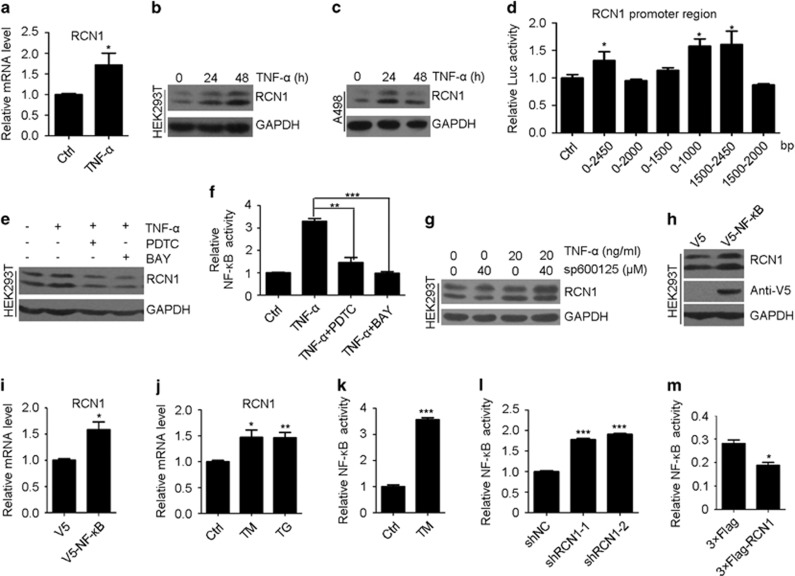
The expression of RCN1 is activated by NF-κB activation. (**a**) Quantitative real-time PCR of relative mRNA expression levels of RCN1 in control (ctrl) and TNF-α (20 ng/ml, 48 h)-treated HEK293T cells. (**b** and **c**) Western blots of RCN1 in control and TNF-α (20 ng/ml, 48 h)-treated HEK293T (**b**) and A498 (**c**) cells. (**d**) Relative luciferase activity of different RCN1 promoter region truncations in TNF-α-treated HEK293T cells. (**e** and **f**) Western blots of RCN1 (**e**) and relative luciferase activity of NF-κB (**f**) in control and TNF-α (20 ng/ml, 48 h)-treated HEK293T cells in the presence or absence of the NF-κB inhibitors pyrrolidine dithiocarbamate (PDTC; 2 μm) or BAY (2 μm). (**g**) Western blots of RCN1 in control and TNF-α (20 ng/ml, 48 h)-treated HEK293T cells in the presence or absence of sp600125. (**h** and **i**) Western blots (**h**) and quantitative real-time PCR of relative mRNA expression levels (**i**) of RCN1 in HEK293T cells transfected with control (V5) and V5-NF-κB. (**j**) Quantitative real-time PCR of relative mRNA expression levels of RCN1 in control (ctrl) and TM (2 μm, 8 h)/TG (1 μm, 8 h)-treated HEK293T cells. (**k**) Relative luciferase activity of NF-κB in control and TM (2 μm, 8 h)-treated HEK293T cells. (**l**) Relative luciferase activity of NF-κB in the negative control (shNC) and RCN1-knockdown (shRCN1) HepG2 cells after treatment with TM (8 μm). (**m**) Relative luciferase activity of NF-κB in control (3 × Flag) and 3 × Flag-RCN1-overexpressing HEK293T cells after TM (2 μm, 8 h) treatment. For **a**, **d**, **f** and **i**–**m**, the data are presented as the mean±s.e.m. *P<0.05, **P<0.01, ***P<0.001, as determined by unpaired two-tailed Student’s *t*-test.

To investigate the upstream mechanism resulting in the upregulation of RCN1 in many cancers,^32^ we focused on TNF-α.^33^ Both mRNA and protein levels of RCN1 in HEK293T and A498 cells increased after TNF-α treatment (Figures 6a and c), thereby suggesting that TNF-α induces RCN1 expression. In addition, luciferase assays revealed that the 0–1000 bp and 1500–2450 bp promoter regions of RCN1 were responsible for its increased expression during TNF-α treatment (Figure 6d). To further examine which pathway downstream of TNF-α regulates the expression of RCN1, we used an inhibitor of c-Jun N-terminal protein kinase (sp600125)^34^ and inhibitors of NF-κB, pyrrolidine dithiocarbamate^35^ and BAY 11-7082 (BAY).^36^ Western blot analysis revealed that the protein level of RCN1 was decreased when the cells were pre-treated with pyrrolidine dithiocarbamate and BAY but was increased after pretreatment with sp600125 (Figures 6e and g). Furthermore, overexpression of an NF-κB subunit also increased RCN1 expression (Figures 6h and i). These data indicate that NF-κB is necessary for the increased expression of RCN1 in the TNF-α pathway.

Given that ER stress activates NF-κB via IRE1 and PERK,^37^ RCN1 probably influences NF-κB activity during ER stress. We observed that the mRNA level of RCN1 increased during ER stress (Figure 6j), and NF-κB activity displayed similar patterns, as detected by luciferase assays (Figure 6k). However, during ER stress, depletion of RCN1 resulted in the activation of NF-κB signaling (Figure 6l), whereas overexpression of RCN1 inhibited NF-κB activation (Figure 6m), thus suggesting a possible regulatory feedback loop between NF-κB and RCN1. Bioinformatics analysis of the TCGA database also revealed that, at the transcription level, RCN1 is positively correlated with NF-κB1 (RELA) and NF-κB2 in all collected cancer samples (Supplementary Figure S6a); these results indicate a positive correlation between RCN1 and NF-κB in cancer. Importantly, high RCN1 mRNA levels were correlated with poor survival rates in kidney clear cell carcinoma, thus suggesting that RCN1 may promote cancer progression (Supplementary Figure S6b).

## Discussion

**Figure 7:**
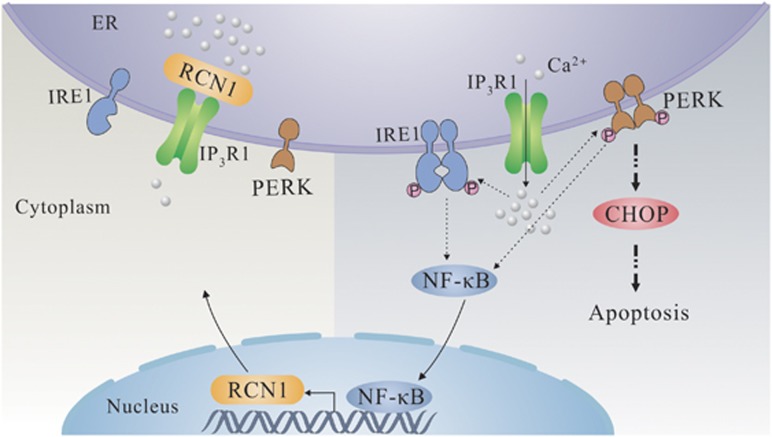
Schematic model. In RCN1 downregulated cells, IP_3_R1 activation disturbs ER calcium homeostasis under conditions of ER stress, thus inducing UPR signaling, such as the PERK–CHOP pathway, and contributing to subsequent apoptosis. In addition, NF-κB, a transcription factor of RCN1, is activated by IRE1 and PERK during ER stress^37^ and then elevates the mRNA level of RCN1, constituting a feedback loop. RCN1 interacts with L3 of IP_3_R1 in a calcium-dependent manner, thereby inhibiting its function and ER calcium release. Therefore, RCN1 suppresses ER stress-induced apoptosis via calcium homeostasis and PERK–CHOP signaling.

RCN1 has previously been reported to be a cancer marker, but its function is largely unknown.^32^ In the present study, we provide evidence that RCN1 interacts with IP_3_R1 in a calcium-dependent manner and inhibits ER calcium release. Under conditions of ER stress, depletion of RCN1 results in disturbances in ER calcium homeostasis, upregulation of the PERK–CHOP signaling pathway and subsequent apoptosis and NF-κB activation. In addition, RCN1 is transactivated by NF-κB and is positively correlated with poor survival in kidney clear cell carcinoma (Figure 7).

Previous reports have suggested that proteins associate with IP_3_R1 and consequently regulate its activity in the ER lumen. Among the known interacting proteins, GRP78 and ERp44 are two critical regulators. GRP78 has been reported to interact with the luminal domain loop 3 (L3) of IP_3_R1 and to stabilize the receptor to increase its function,^38^ whereas ERp44 competes with GRP78 for the same interaction region on IP_3_R1 and inhibits its activity and ER Ca^2+^ depletion.^39^ Here, we report that RCN1, an ER luminal protein, also interacts with L3 of IP_3_R1, thereby inhibiting ER calcium release. These results indicate that RCN1 may have important roles in ER calcium homeostasis. However, the specific relationship among RCN1, ERp44 and GRP78 requires further investigation.

Numerous calcium-binding proteins target the cytosolic region of IP_3_Rs. For example, calmodulin, a cytosolic calcium-binding protein, binds to the N-terminal region of IP_3_R1 and inhibits its activation.^40^ CaBP, a Ca^2+^ binding protein, has been shown to activate IP_3_R1 in the absence of IP_3_.^41^ Furthermore, some calcium-binding proteins act as calcium sensors. STIM1, an ER transmembrane protein, senses decreased calcium concentrations in the ER lumen, changes its conformation and consequently activates the calcium channel ORAI.^42^ A previous study has confirmed that RCN1 levels are altered at different calcium concentrations,^29^ and our data indicate that as ER calcium concentrations decrease below normal levels, the interaction of RCN1 and IP_3_R1 increases, thus suggesting that during ER stress, RCN1 may sense the decrease in [Ca^2+^]_ER_ and subsequently bind to IP_3_R1 and prevent further ER calcium depletion. Therefore, RCN1 may act as a calcium sensor during ER stress.

Because of its C-terminal HDEL ER retention signal, RCN1 is localized to the ER lumen in most cells.^32^ However, plasma membrane distribution of RCN1 has been observed in bone endothelial cells and prostate cancer cells and has been found to increase after treatment with tumor necrosis factor α (TNF-α).^33^ Here, we report that the mRNA and protein levels of RCN1 increase after treatment with TNF-α, which may override the ability of KDEL receptors to transport proteins with ER retention signals to the ER lumen^43^ and result in increased RCN1 membrane distribution. However, the detailed mechanisms underlying these observations remain unclear.

Previous reports have demonstrated that increased RCN1 expression is associated with inflammation in epithelial and non-epithelial cells, but the detailed mechanism is ambiguous.^44^ NF-κB, one of the major signaling pathways downstream of TNF-α, is a key regulator of the onset of inflammation.^45^ Here, we report that NF-κB upregulates the expression of RCN1 by activating its promoter region, thus partially explaining the relationship between RCN1 and inflammation.

During ER stress, NF-κB can be activated by multiple events. For instance, activated IRE1 may bind to the TNF-α receptor associated factor 2 complex, which then recruits IκB kinase which in turn phosphorylates and degrades IκB and consequently promotes NF-κB activation.^46^ Here, our data indicate that depletion of RCN1 increases NF-κB signaling and activates IRE1 and PERK. However, only PERK participates in the apoptosis process, whereas IRE1 exhibits no effect. Thus, the function of IRE1 activation remains unclear. However, the possibility that RCN1 regulates apoptosis in a calcium-independent manner cannot be excluded. Given that NF-κB increases the expression of RCN1, there may exist a feedback mechanism such that downregulation of RCN1 leads to the activation of IRE1 and PERK, thus increasing NF-κB signaling and stimulating the cell to increase RCN1 expression and to restore homeostasis. In addition, on the basis of our bioinformatics analysis, RCN1 is correlated with poor survival in kidney clear cell carcinoma, thus suggesting that RCN1 may be a potential target for cancer therapy.

## Materials and methods

### Vector construction

RCN1 cDNA was cloned from HeLa cell cDNA and inserted into a pcDNA3.1+ vector (Invitrogen, Carlsbad, CA, USA) with a 3 × Flag tag immediately after its signal peptides. RCN1 EF-hand deletion mutants and truncation mutants with the 3 × Flag tag were constructed on the basis of the vectors described above. IP_3_R1 truncation cDNAs were cloned from HeLa cell cDNA and inserted into a pEGFP-N3 vector (BD Biosciences, San Jose, CA, USA).

The following short hairpin RNA (shRNA) sequences were used: RCN1 sh1, 5′-CCGCAGAGTTTCATGATTCTT-3′ and sh2, 5′-AGAAGCTAACTAAAGAGGAAA-3′ (Sigma, St Louis, MO, USA); PERK sh1, 5′-GAAATACTCTACCAGCCTCTA-3′ and sh2, 5′-GAAACAGCTATTCTCATAAAG-3′ (Sigma); IRE1 sh1, 5′-GAGAAGATGATTGCGATGGAT-3′ and sh2, 5′-GAAATACTCTACCAGCCTCTA-3′; IP_3_R1 siRNA1, 5′-GAGAAUUUCCUUGUAGACAUCUGCA-3′ and siRNA2, 5′-GGCCUGAGAGUUACGUGGCAGAAAU-3′ and CHOP siRNA1, 5′-UGGGAGAACCAGGAAACGGAA-3′ and siRNA2, 5′-AAGGAAGUGUAUCUUCAUACA-3′.

### Reagents and antibodies

Primary antibodies included anti-RCN1 (Bethyl, Montgomery, TX, USA), anti-GFP (MBL, Nagoya, Japan), anti-Flag (Sigma), anti-IP_3_R1 (Bethyl), anti-tubulin (Sigma), anti-ATF4 (GeneTex, San Antonio, TX, USA), anti-pIRE1 (Abcam, Cambridge, MA, USA), anti-pPERK, anti-caspase-3, anti-PERK, anti-IRE1, anti-GAPDH, anti-CHOP, anti-eIF2α and anti-GRP78/BiP (Cell Signaling Technology, Danvers, MA, USA). The drugs used were TG (Sigma), TM (Sigma), sp600125 (Cell Signaling), 2-APB (Sigma), Xec, pyrrolidine dithiocarbamate, BAY 11-7082, GSK2606414 and STF883010 (Selleckchem, Houston, TX, USA).

### Cell culture and transfection

HEK293T, HEK293, HepG2,^47^ B16F10 and A498 cells were cultured in Dulbecco’s Modified Eagle’s medium (GIBCO BRL, Grand Island, NY, USA) supplemented with 10% fetal bovine serum (HyClone, Logan, UT, USA or CellMax) at 37 °C and 5% CO_2_. The cells were transfected with polyethyleneimine when they reached 50% confluency. Five hours after transfection, the medium was replaced, and cells were cultured with or without drugs for the indicated times and were used for subsequent study.

### Stable cell line generation

HepG2, HEK293T and B16F10 cells were transfected with plasmids and then incubated with puromycin (2 μg/ml) for 1 week. Subsequently, single clones were selected and cultured in the presence of puromycin for 2 weeks. The cell lines were then subjected to western blot analysis.

### Quantitative real-time PCR

The mRNAs were extracted from HeLa cells to synthesize cDNA using a GoScript Reverse Transcription System (Promega, Madison, WI, USA). SYBR Green PCR Master Mix (Applied Biosystems, Foster City, CA, USA) was used to perform quantitative real-time PCR in an ABI 7300 Detection System (Applied Biosystems) as previously described.^48^ The primer sequences are listed in Supplementary Materials (Supplementary Table S1). All the reactions were performed in triplicate.

### Immunoprecipitation, *in vitro* binding assays and GST pull-down assays

The HEK293T cells were collected after transient transfection with vectors for 36–48 h. The cells were lysed in immunoprecipitation buffer (25 mM HEPES, 150 mM NaCl, 1% Triton X-100, 1 mM DTT (dithiothreitol), 2 mM CaCl_2_, 1 mM MgCl_2_, pH 7.4) containing a protease inhibitor cocktail (Roche, Indianapolis, IN, USA). One-tenth of the cell lysates was used as input samples, and the rest was pre-cleared with Protein A/G Sepharose beads (GE Healthcare, Uppsala, Sweden) for 0.5 h. The cell lysates were incubated with primary antibody at 4 °C overnight, after which the beads were added to the system and incubated for 2 h. The beads were then rinsed in the same immunoprecipitation buffer six to eight times and were collected and subjected to western blot analysis.

For *in vitro* binding assays in different Ca^2+^ concentrations, HEK293T cells 48 h post-transfection were lysed in immunoprecipitation buffer (25 mM HEPES, 150 mM NaCl, 1% Triton X-100, 1 mM DTT, 1 mM MgCl_2_, pH 7.4), and the total cell lysates were separated. The Ca^2+^ concentration was adjusted using a CaCl_2_ solution, and the solution was incubated with primary antibody at 4 °C overnight.

### MTS assay

The cells were seeded in 96-well plates at 5 × 10^3^ per well, treated with drugs at the indicated times after transfection, and assayed using an MTS kit (Promega). Before measurement, the medium was replaced, and 20 μl of MTS reagent was added to each well. After 1–2 h of incubation, relatively viable cells were measured as the absorbance at 490 nm. Each experiment was repeated eight times.

### Calcium imaging

The cells were loaded with fluo-4 AM (Invitrogen) at 4 mg/ml in buffer (140 mM NaCl, 5 mM KCl, 1 mM MgCl_2_, 10 mM glucose, 10 mM HEPES, pH 7.35) for 20 min at 37 °C in the dark. After loading, the cells were washed using the same buffer without fluo-4. The cells were excited at 480 nm, and the fluorescence emission at 505 nm was recorded. The data were analyzed using GraphPad Prism software.

### Flow cytometry analysis

After transfection or treatment with drugs, the cells were collected and re-suspended in annexin V-binding buffer, and the cell density was adjusted to 10^6^ cell/ml. Approximately 2 μl of annexin V and 1 μl of 100 μg/ml PI was added to the cell suspension, and this was followed by incubation in the dark at RT for 20 min. The stained cells were analyzed by flow cytometry, and the fluorescence emission at 530 nm and 648 nm was measured.

### TUNEL assays

After treatment with drugs, the cells were subjected to TUNEL labeling according to the recommended procedures (*In Situ* Cell Death Detection kit, TMR red; Roche). Briefly, the cells were fixed in 4% paraformaldehyde for 30 min and then treated with 0.1% Triton X and 0.1% sodium citrate (freshly prepared) on ice for 2 min. After being washed with phosphate-buffered saline, the cells were loaded with 50 μl of reaction mixture (enzyme solution 5 μl and label solution 45 μl) for 60 min at 37 °C in the dark.^49^

### Data analysis

All experiments were repeated at least three times. Data analysis was performed with GraphPad Prism 5 software (GraphPad Software) using unpaired two-tailed Student’s *t*-tests.
