# Supplementary material for: RCN1 suppresses ER stress-induced apoptosis via calcium homeostasis and PERK–CHOP signaling
Source: Oncogenesis. 2017 Mar 20;6(3):e304–. doi: 10.1038/oncsis.2017.6 (PMC5533947; doi:10.1038/oncsis.2017.6)
Supplement: Supplementary Information [file oncsis20176x1.docx]

**Supplemental Information**

**RCN1 suppresses ER stress-induced apoptosis via calcium homeostasis and PERK-CHOP signaling**

Sizheng Xu, Yiwei Xu, Liang Chen, Qiao Fang, Sixian Song, Jianguo Chen, and Junlin Teng

**Supplemental Figure Legends**

**Supplementary Figure S1.** RCN1 inhibits ER stress-induced apoptosis. (a) Phase contrast microscopy of TM-treated control or RCN1-knockdown HEK293T cells. (b) Representative images from TUNEL assays of labeled apoptotic cells in control or RCN1-knockdown HepG2 cells treated with TM (8 μM) for 36 h. Scale bar, 50 μm. (c and d) Western blots of cleaved caspase-3 in RCN1-knockdown HepG2 cells (c) and HEK293T cells (d). (e) Flow cytometry analysis of apoptotic cells labeled by annexin Ⅴ and PI in TG (4 μM, 36 h)-treated control (shNC) or RCN1-knockdown HEK293T cells. NC, negative control. (f) Flow cytometry analysis of apoptotic cells labeled by annexin Ⅴ and PI in control or RCN1-knockdown HEK293T cells. (g) Quantification of apoptotic cells as in (f**)** (n=3; 10000 cells per experiment). (h) Flow cytometry analysis of apoptotic cells labeled by annexin Ⅴ and PI in TG (1 μM, 36 h)-treated control (3×Flag) or RCN1-overexpressing HEK293T cells. (i) Flow cytometry analysis of apoptotic cells labeled by annexin Ⅴ and PI in control (3×Flag) or RCN1-overexpressing HEK293T cells. (j) Quantification of apoptotic cells as in (i**)** (n=3; 10000 cells per experiment). (k) Western blots of cleaved caspase-3 in control (3×Flag) or RCN1-overexpressing HEK293T cells.

**Supplementary Figure S2.** RCN1 inhibits UPR signaling. (a) Quantitative real-time PCR of the relative mRNA expression levels of p58^ipk^, Herp, ERdj4, ATF4, EDEM1, and WARS in the negative control (shNC) and RCN1-knockdown HepG2 cells after treatment with TM (2 μM). (b) Western blots of cleaved ATF6 in control and RCN1-knockdown HepG2 cells after treatment with TM (8 μM) for 6 h. (c-e) Quantitative real-time PCR of the relative mRNA expression levels of Xbp1s (c), GRP78 (d), and CHOP (e) in 3×Flag- and 3×Flag-RCN1-overexpressing HEK293T cells after TG (1 μM, 8 h) treatment. For (a), (c), (d) and (e), data are presented as the mean ± SEM. n.s. indicates no significant difference, **P* < 0.05, ***P* < 0.01, ****P* < 0.001, as determined by unpaired two-tailed Student’s *t*-test.

**Supplementary Figure S3.** RCN1 inhibits ER stress-induced apoptosis via PERK-CHOP signaling. (a) Flow cytometry analysis of apoptotic cells labeled by annexin Ⅴ and PI after TM (8 μM) treatment in the presence or absence of STF083010 (2 μM) treatment in the negative control (shNC) and RCN1-knockdown (shRCN1) HepG2 cells. (b) Quantification of early apoptotic cells as in (a) (n=3; >100 cells per experiment). Data are presented as the mean ± SEM. ****P* < 0.001, n.s. no significant difference, as determined by unpaired two-tailed Student’s *t*-test. (c) Western blots of IP_3_R1-knockdown efficiency in control and RCN1-knockdown HepG2 cells.

**Supplementary Figure S4.** RCN1 interacts with IP_3_R1. (a) HEK293T cells co-transfected with 3×Flag-RCN1 and GFP, IP_3_R1-TM (transmembrane domain)-GFP or IP_3_R3-TM-GFP were subjected to immunoprecipitation (IP) using an anti-GFP antibody. The immunoprecipitates were immunoblotted with an anti-Flag or anti-GFP antibody. (b) Schematic of RCN1 truncation constructs. SP, signal peptide; EFh, EF-hand. (c) HEK293T cells co-transfected with IP_3_R1-TM-GFP and 3×Flag, 3×Flag-RCN1, or 3×Flag-RCN1-DEL-EFh1~6 were subjected to immunoprecipitation using an anti-Flag antibody. The immunoprecipitates were immunoblotted with an anti-Flag or anti-GFP antibody. (d) HEK293T cells co-transfected with 3×Flag-RCN1 and IP_3_R1-TM-GFP were subjected to immunoprecipitation using an anti-GFP antibody in IP buffer at the indicated calcium concentrations. The immunoprecipitates were immunoblotted with an anti-Flag or anti-GFP antibody.

**Supplementary Figure S5.** RCN1 inhibits apoptosis via IP_3_R1. (a) Quantification of early apoptotic cells labeled by annexin Ⅴ and PI after TG (4 μM) treatment in the presence or absence of Xec (0.1 μM) or 2-APB (2 μM) pretreatment in the negative control (shNC) or RCN1-knockdown (shRCN1) HepG2 cells in a flow cytometry analysis. (b) Quantification of early apoptotic cells labeled by annexin Ⅴ and PI after TG (1 μM) treatment in the presence or absence of Xec (0.1 μM) or 2-APB (2 μM) pretreatment in control (3×Flag) or 3×Flag-RCN1-overexpressing HepG2 cells determined by flow cytometry analysis.

**Supplementary Figure S6.** The transcription level of RCN1 is positively correlated with NF-κB and poor survival in cancer. (a) Correlation of the mRNA levels of RCN1 and NF-κB1 (RELA) or NF-κB2 in all collected cancer samples of TCGA. (b) Overall survival of different groups of kidney clear cell carcinoma patients. The high and low expression groups were divided at the median of the RCN1 mRNA level. For (a), data were determined by unpaired two-tailed Student’s *t*-test; for (b), data were determined by Pearson correlation analysis.

**Supplementary Table 1** **Primer information for quantitative real-time PCR.**

| Gene | Sense | Antisense |
| --- | --- | --- |
| ATF4 | CAGCACAGCCCCTCTACCA | GCCCGCCTTAGCCTTGTC |
| WARS | ACAAGGGGAGCTCGTAAGGT | TATGCCTTTTGCACTGCTTG |
| CHOP | AAGGAAAGTGGCACAGCTAGCT | CTGGTCAGGCGCTCGATTT |
| EDEM1 | AACCTCCGGAGCAATGATACAG | CAGTGGCCCGTCTGAATGT |
| ERdj4 | CATCAGAGCGCCAAATCAAG | TTTTTGTCAGGGTGGTACTTCATG |
| GRP78 | TGGCGGAACCTTCGATGT | GCCACAACTTCGAAGACACCAT |
| GAPDH | GGCATCCTGGGCTACACTGA | GGAGTGGGTGTCGCTGTTG |
| XBP1s | CCGGTCTGCTGAGTCCGCAGC | TGGCAGGCTCTGGGGAAGGG |
| p58^IPK^ | GAGGTTTGTGTTGGGATGCAG | GCTCTTCAGCTGACTCAATCAG |
| Herp | CCAAGGCCTGGGGCCTGG | CTGTCGAGTCCACGCCAGG |
