# Supplementary figures and images for: RCN1 suppresses ER stress-induced apoptosis via calcium homeostasis and PERK–CHOP signaling
Source: Oncogenesis. 2017 Mar 20;6(3):e304–. doi: 10.1038/oncsis.2017.6 (PMC5533947; doi:10.1038/oncsis.2017.6)

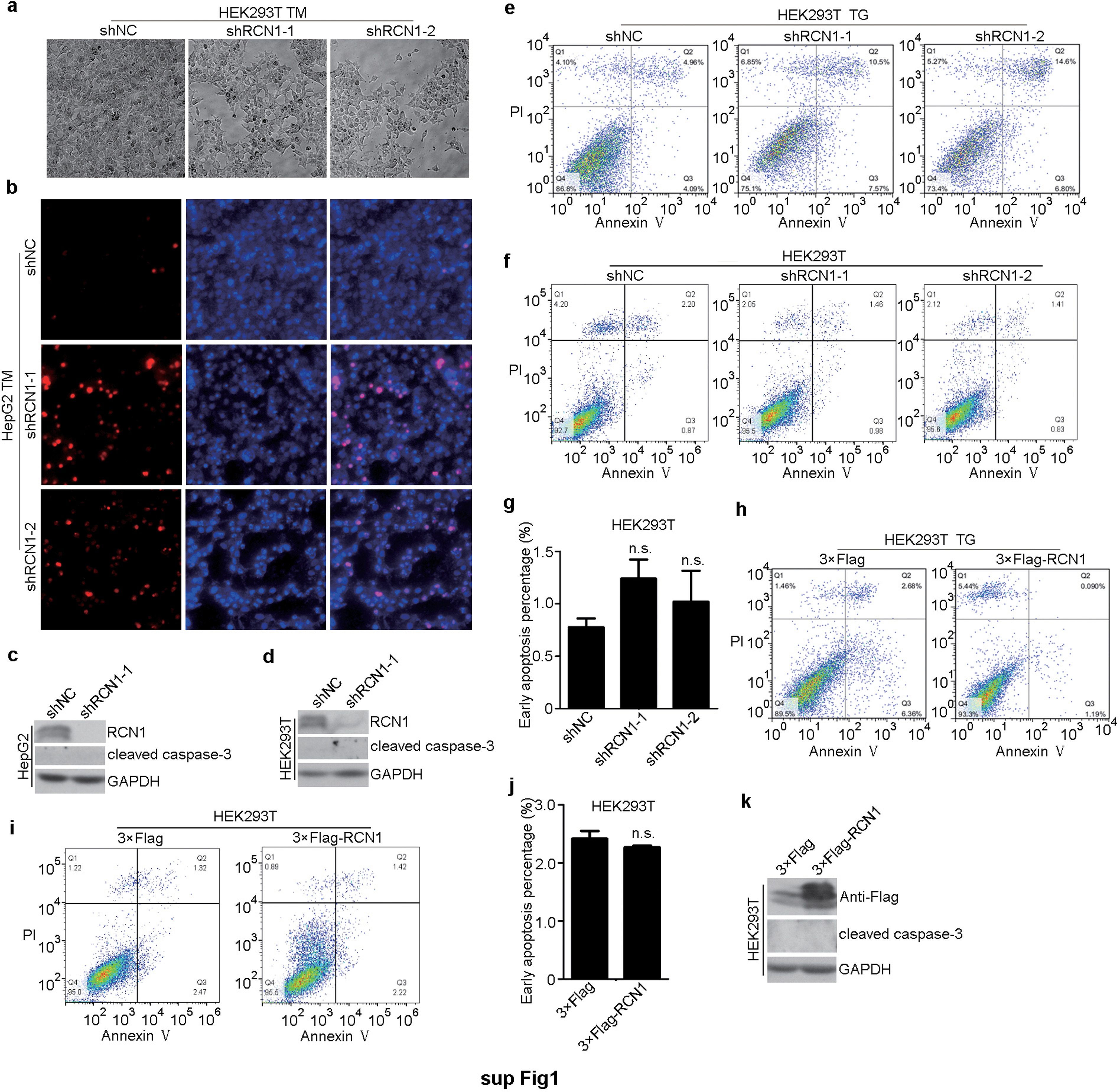

Supplement: Supplementary Figure 1 [file oncsis20176x2.tif]

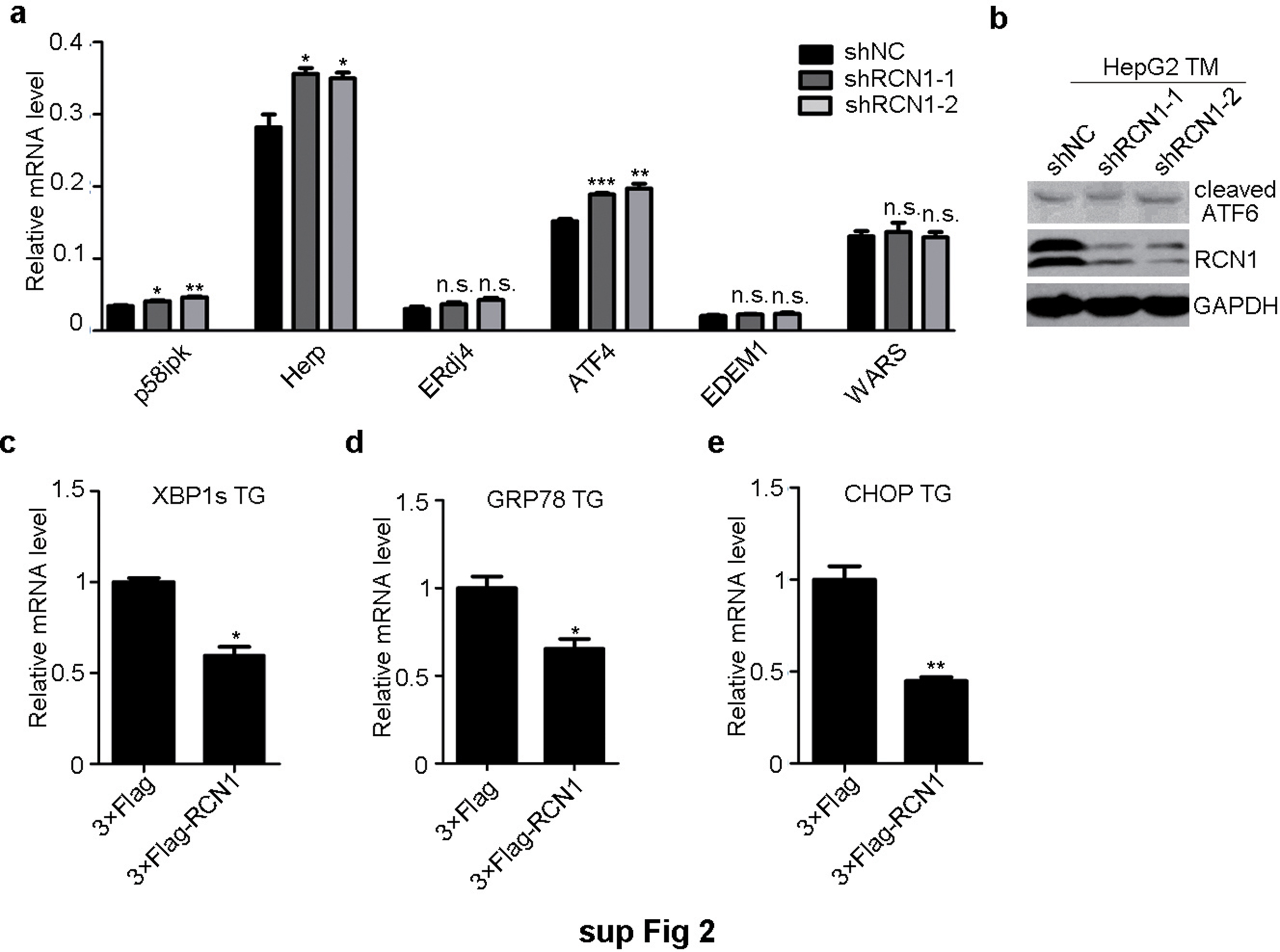

Supplement: Supplementary Figure 2 [file oncsis20176x3.tif]

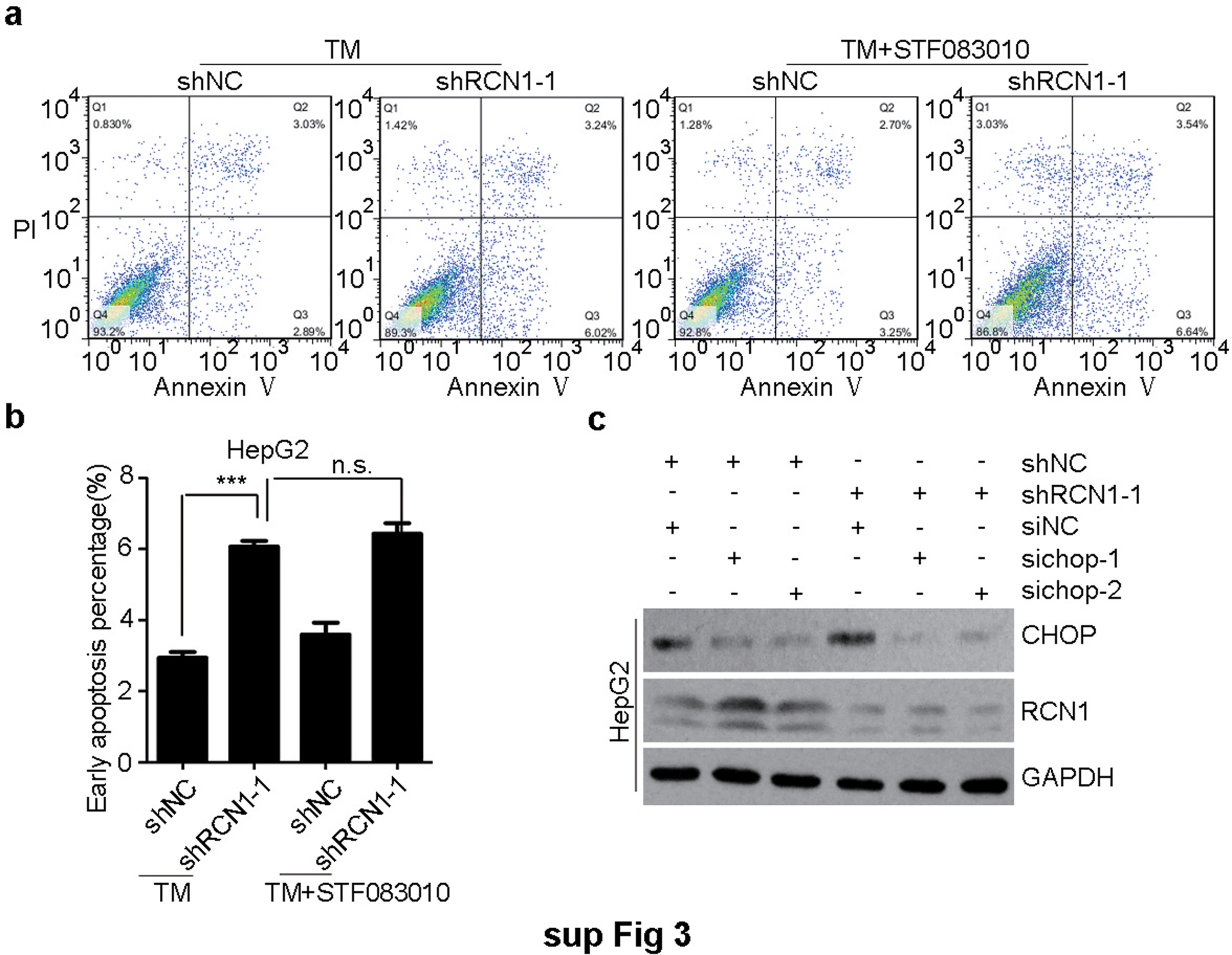

Supplement: Supplementary Figure 3 [file oncsis20176x4.tif]

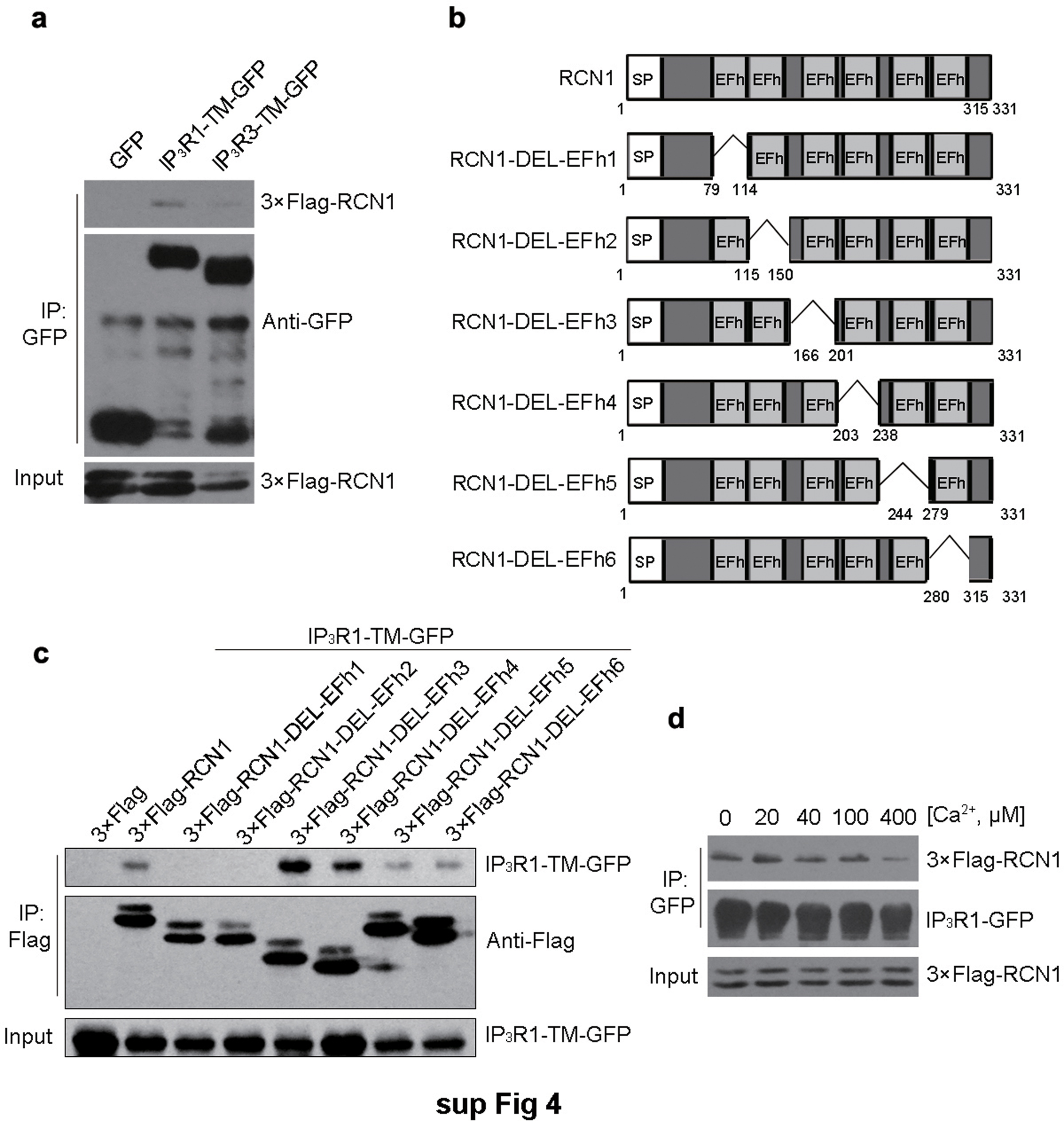

Supplement: Supplementary Figure 4 [file oncsis20176x5.tif]

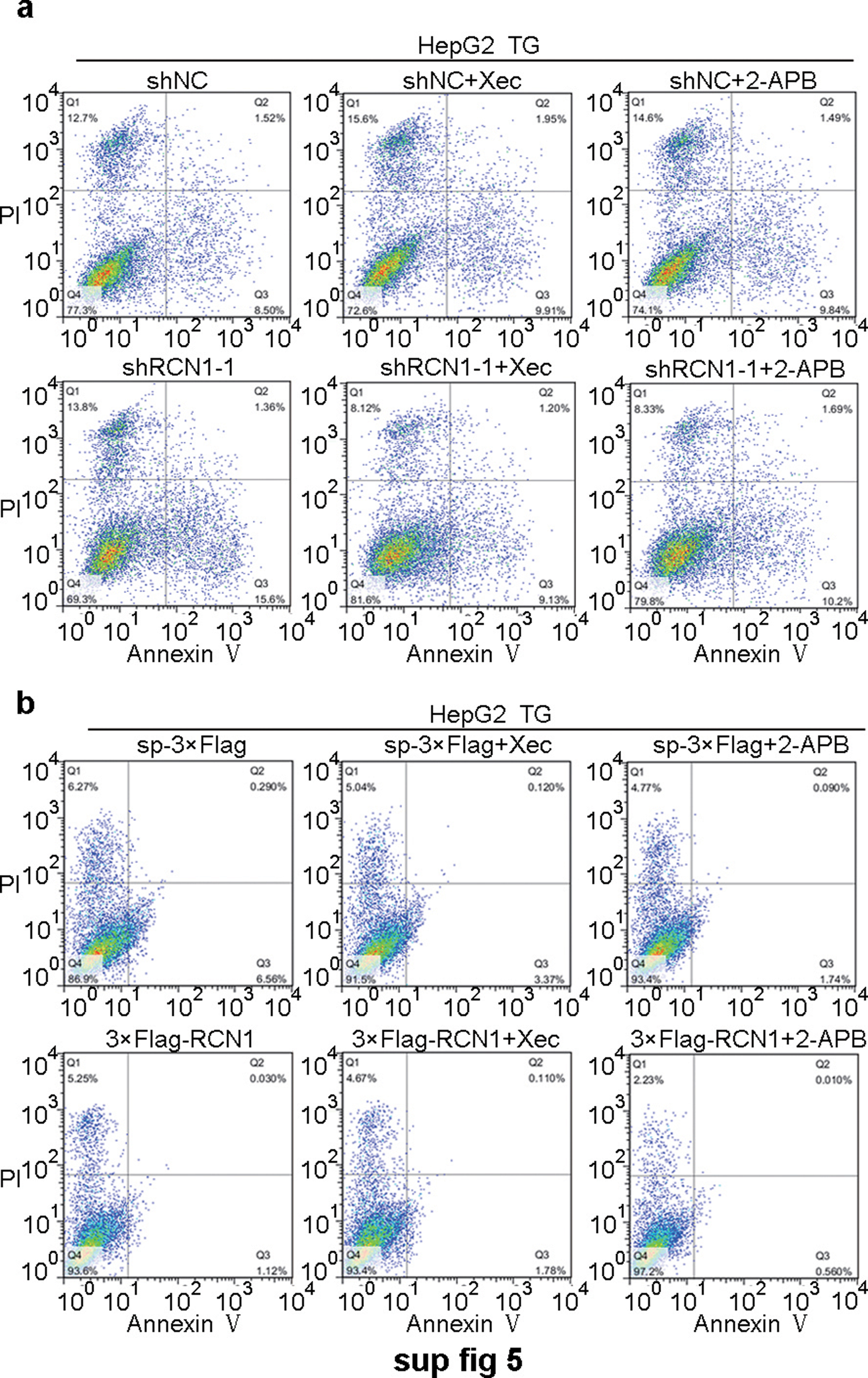

Supplement: Supplementary Figure 5 [file oncsis20176x6.tif]

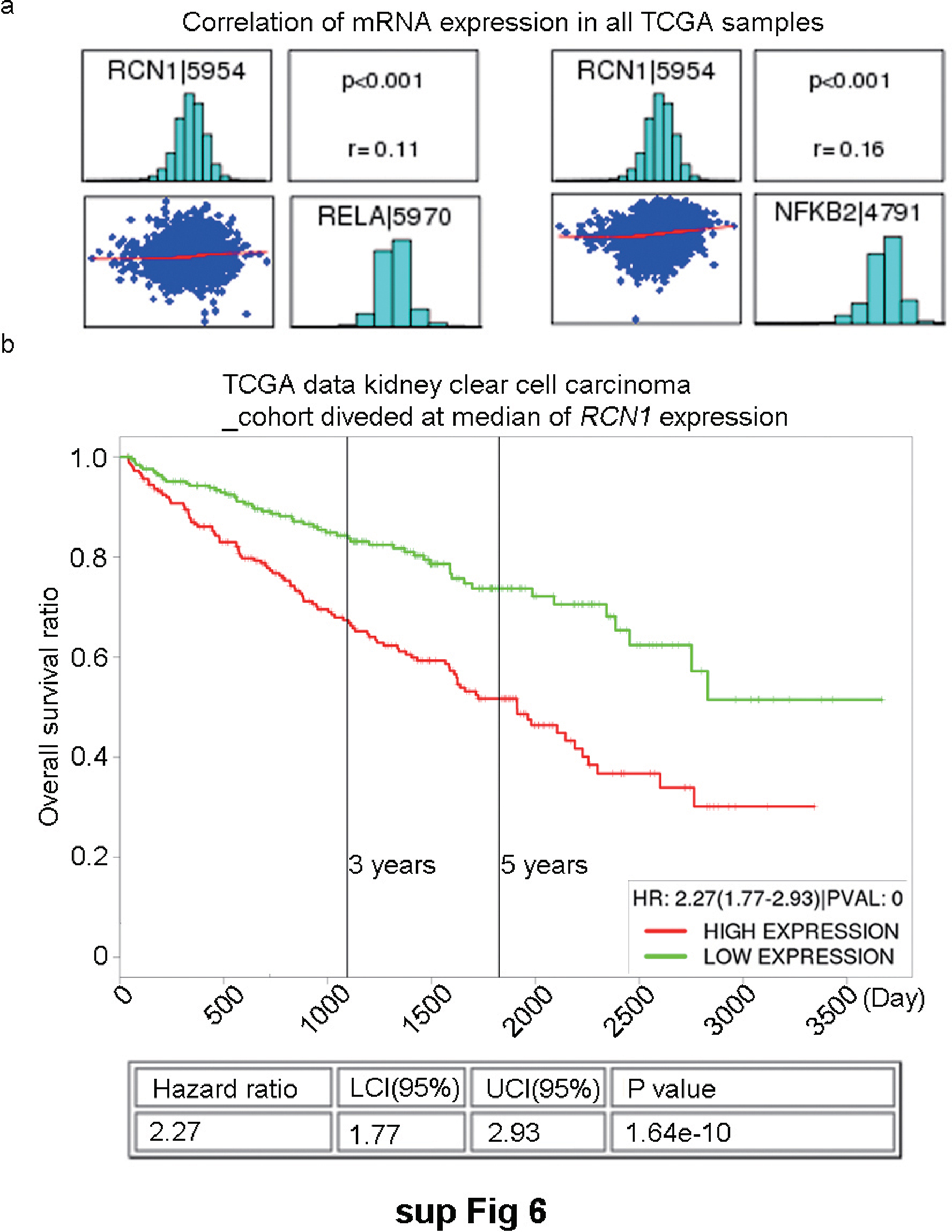

Supplement: Supplementary Figure 6 [file oncsis20176x7.tif]
